# Diagnostic Concordance between Random Upper Arm Circumference and Mid Upper Arm Circumference Measurements among Children Aged 6–59 Months in South Ethiopia: A Community-Based Cross-Sectional Study

**DOI:** 10.1155/2021/6654817

**Published:** 2021-03-31

**Authors:** Juhar Admama Bamud, Afework Mulugeta Bezabih, Fentaw Wassie Feleke, Getahun Fentaw Mulaw

**Affiliations:** ^1^South Nations and Nationalities Peoples Regional State, Silte Zonal Health Department, Worabe, Ethiopia; ^2^College of Medicine and Health Science, Mekelle University, Mekelle, Ethiopia; ^3^School of Public Health, College of Health Science, Woldia University, Woldia, Ethiopia

## Abstract

Random upper arm circumference (RUAC) is frequently used for malnutrition screening among children aged 6–59 months. However, inadequate evidence exists regarding its agreement with mid upper arm circumference (MUAC). This study examined diagnostic concordance between RUAC and standard MUAC measurements and tested RUAC's ability for screening malnutrition among children aged 6–59 months. A cross-sectional study was conducted from April 30 to May 30/2015 in Ethiopia. Data were taken from a sample of 819 children aged 6–59 months with a simple random sampling technique. The data were analyzed using SPSS version 22 software. The kappa agreement level, sensitivity, and specificity were calculated. The receiver operating curve was prepared to determine the optimal cutoff RUAC for the sensitivity and specificity. With National Center for Health Statistics (NCHS) cutoff (12 cm), the performance of RUAC measurements in terms of sensitivity was low (44%). The kappa agreement level between the two measurements was 0.551 (*p* value < 0.001). With the new WHO cutoff (12.5 cm), however, RUAC was improved in validity (sensitivity 81%), specificity 96.9%, and kappa agreement level (*K* = 0.807; *p* < 0.001).

## 1. Introduction

Nutrition is a core pillar of human development and concrete; large-scale programming not only can reduce the burden of undernutrition and deprivation but also advances the progress of nations. Reliable data on child growth are a prerequisite for monitoring and improving child health. Despite the extensive resources invested in recording anthropometry, there has been little research on the validity of these data. If these measurements are not valid, growth may be misreported, and health problems may go undetected [1]. In countries like Ethiopia, it is specifically more important as 9% of children aged less than five years suffer from wasting due to acute undernutrition, 25% of Ethiopian children less than five years are underweight, and 40% are stunted due to chronic undernutrition [2].

The MUAC and the weight for height (WHZ) are the two anthropometric indicators most often used to identify children with acute malnutrition. The WHZ < −3 using the 2006 WHO Growth Standards is used to identify malnutrition deficits in tissue and fat mass compared with the amount expected in a child of the height or length. It is a sufficient tool for screening in emergencies which is counting for undernourished [3]. But the concept underlying their use as a screening tool for SAM differs. For so many years, MUAC has been used as a prescreening tool to identify wasted children, and WHZ is used as a confirmatory test [4].

The MUAC is gaining popularity over WH-based diagnosis as a tool to follow up children during nutrition rehabilitation [5, 6]. This is because, MUAC has only one measurement, while WH needs two, it uses lighter and cheaper materials, and no reference tables are required (unlike WH-based diagnosis) [6, 7]. In peripheral health facilities or in the community, where height is not easily measured, the circumference of the upper arm can be used in place of the WH *z-*score. But it changes little between the children aged 6 and 59 months [8]. The development of therapeutic care for acute malnutrition at the community level necessitated the need for MUAC because it was easier than WH [9]. The MUAC has been routinely used as a tool for nutrition screening in preschool children by government agencies and nongovernmental organizations promoting nutrition and child health interventions [10].

According to WHO and MUAC, cutoff of 115 mm has been recommended to gain sensitivity in detecting severely wasted children [11]. But several countries, such as Ethiopia, use MUAC < 110 mm as the cutoff for admission to services to manage severe acute malnutrition and MUAC < 120 mm as the cutoff for admission to services [12]. However, there has been a lot of controversy over whether MUAC is age and sex independent in under five years children [13].

The RUAC where the midpoint of the upper arm is randomly selected as opposed to MUAC where it is measured at the midpoint between the tip of the shoulder and the tip of the elbow is routinely used to screen children for their nutritional status in Southern Ethiopia. However, there is a paucity of data on the diagnostic ability of RUAC and its implications to identify malnourished children. Doubts have also been raised by different practitioners about the diagnostic validity of RUAC. This study was thus aimed at examining diagnostic concordance between the RUAC and standard the MUAC measurements and testing the RUAC's ability for screening acute malnutrition in children aged 6–59 months.

## 2. Materials and Methods

### 2.1. Study Settings

This study was conducted in Silte Zone, Lanfro District, purposively. Lanfro District is one of the 8 districts and one city administration in Silte Zone, located at about 270 kilometers west of Hawassa, the capital of SNNPR, and 184 km from Addis Ababa. The district borders are Silte District in the north, Oromia Region in the east, Silte and Dalocha Districts in the west, and Halaba Special District in the south. The district is divided into 25 rural and two urban kebeles. The district is characterized by one agro-ecological zone which is kola/low land.

The total population of the district is estimated at 138,839 in the study period. There are about 19437 children aged 6–59 months in this district. The population density is 345/km^2^ and the average landholding size is more than 0.5 ha/household. Regarding ethnicity, the majority are Silte. The dominant religion is Muslim. The population is dependent on mixed subsistence farming. Maize, wheat, and teff are the main food crops with wheat being the main cash crop and maize being the major perennial crop used for consumption. The staple foods in the area are “wheat” and “maize.”

### 2.2. Study Period

Data were collected from April 30/2015 to May 30/2015.

### 2.3. Study Design

A tool validation study was conducted based on data collected through a community-based cross-sectional study design among children aged 6–59 months in Lanfro District, Silte Zone, and SNNPR.

### 2.4. Source Population

All children aged 6–59 months are from the Lanfro District.

### 2.5. Study Population

Children aged 6–59 months are from the six kebeles of the district.

### 2.6. Inclusion Criteria

All children aged between 6 and 59 months and residents of the study area for at least 6 months were included in the study.

### 2.7. Exclusion Criteria

Seriously ill children and those with physical disabilities that make it difficult to take MUAC and RAUC measurements were excluded from the study.

### 2.8. Sample Size Determination

The adequate power of the study depends on the sample size. Power is a probability that a statistical test will indicate a significant difference where a certain prespecified difference is actually present. In a survey of eight leading journals, only two out of 43 studies reported a prior calculation of sample size in diagnostic studies [14]. In estimation setup, adequate sample size ensures that the study will yield the estimate with desired precision. A small sample size produces imprecise or inaccurate estimate, while a large sample size is wastage of resources especially when the test is expensive. The sample size was calculated using Buderer's formula at the required absolute precision level for sensitivity and specificity [15]:

(1) $$\begin{array}{c} \text{sample size}\left(n\right)\text{based on sensitivity}=\frac{{\left(Z1-\alpha /2\right)}^{2}\times \text{SN}\times \left(1-\text{SN}\right)}{L^{2}\times \text{prevalence}},\\ \text{sample size}\left(n\right)\text{based on specificity}=\frac{{\left(Z1-\alpha /2\right)}^{2}\times \text{SP}\times \left(1-\text{SP}\right)}{L^{2}\times \left(1-\text{prevalence}\right)}. \end{array}$$

Here, *n* denotes the required sample size, SN denotes the anticipated sensitivity of MUAC which was assumed to be 85% (since MUAC is a screening criterion, better use SN > 80%), SP denotes the anticipated specificity of MUAC which was assumed to be 96% (since MUAC is a screening criterion, SP > 80%), *α* is the size of the critical region (1 − *α* is the confidence level), *Z*1 − (*α*/2) is the standard normal deviate corresponding to the specified size of the critical region (*α*) = 1.96, *L* is the absolute precision desired on either side (half-width of the confidence interval) of sensitivity or specificity which is 0.05, and nonresponse rate is 10%. The prevalence of undernutrition in children in SNNPR was 0.263 [2].

(2) $$\begin{array}{c} \text{sensitivity}=\frac{1.96\times 1.96\times 0.85\times \left(1-0.85\right)}{0.05\times 0.05\times 0.263}+10\%\text{ NRR},\\ n=819,\\ \text{specificity}=\frac{1.96\times 1.96\times 0.96\times \left(1-0.96\right)}{0.05\times 0.05\times \left(1-0.263\right)}+10\%\text{ NRR},\\ n=88. \end{array}$$

As can be seen from the sample size calculations for sensitivity and specificity, the largest possible sample size was 819.

### 2.9. Sampling Technique and Procedure

**Figure 1:**
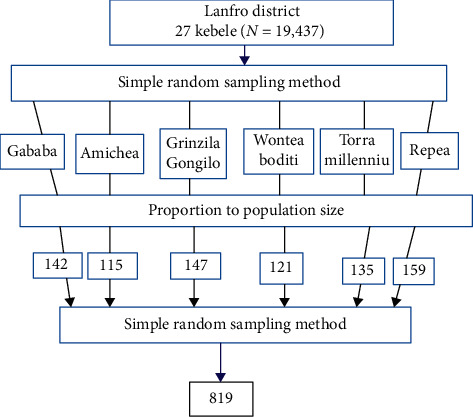
Schematic presentation of the sampling procedures on diagnostic concordance between RUAC and MUAC among children aged 6–59 months in south Ethiopia.

From the total of 27 kebeles of Lanfro District, only six kebeles were selected by simple random sampling (lottery method). After the households with the eligible age groups of children (between 6 and 59 months old) were identified from the family folder and recent community health day (CHD) registration book in the health post, a list was prepared and that was used as a sampling frame. Study subjects were allocated to each kebele based on probability proportional to size (PPS) (Figure 1). By using ENA SMART 2011, a random table was generated to select the eligible households. When more than one child is found in the selected household, the youngest child was selected for this study. If young twins were found within the selected household, a child was taken through the simple random sampling lottery method.

### 2.10. Data Collection Methods

Anthropometric data were collected using a structured questionnaire by 12 trained diploma clinical nurses who have experience in screening malnutrition from the health center.

The age of the children was collected by the health extension workers from the child family folder. Child family folder is a blue folder kept at health after delivery. It contains important details of the child including age, sex, and health status, and this is updated each time when the mother comes for a postnatal visit and immunization session. Mothers who did not have the child's family folder deduced the age by the use of local event calendars.

### 2.11. MUAC Measurement

MUAC was measured on the left arm of all sampled under five children following the standard techniques of MUAC measurement as recommended in the 2008 revised interim guideline for emergency nutrition assessment for Ethiopia [16]. The left arm was flexed to 90 degrees at the elbow after locating and marking the midpoint between the lateral acromion and distal olecranon of the upper arm. The arm was relaxed, the MUAC strip was placed comfortably around the marked midpoint of the arm, and the measurement was recorded to the nearest 0.1 cm.

### 2.12. RUAC Measurement

The RUAC where the midpoint of the upper arm is randomly selected as opposed to MUAC where it is measured at the midpoint between the tip of the shoulder and the tip of the elbow is routinely used to screen children for their nutritional status. RUAC measurements were made on the child's left arm by trained data collectors according to usual measurement of health workers to screen acute malnutrition. Measurement was recorded to the nearest 0.1 cm. RUAC measurement was taken twice from the same child.

### 2.13. Data Quality Control

To ensure data quality, training was given to 12 diploma nurse data collectors and 3 supervisors. Close supervision during the data collection period was conducted by supervisors. Demonstration of MUAC and RUAC technique in front of the trainer and review of potential sources for error were done. RUAC was first measured to minimize bias raised from the marked point of the child's left upper arm for MUAC measurement. RUAC and MUAC measurements were measured by the same measurer in order to minimize measurement error between different measurers. MUAC was not measured immediately after RUAC on the same child to avoid bias which might arise from the fresh memory of the values of the RUAC measurement. Trainees were allowed to practice taking MUAC and RUAC measurements on each other. The questionnaire was pretested in communities of similar setting and was adjusted accordingly. The questioner was first developed in English and translated to the Amharic language.

### 2.14. Statistical Analysis

Epi Data entry version 3.1 was used to manage the data quality and then exported to SPSS version 22 software for further analysis. The extent of the agreement between MUAC and RUAC was determined by using kappa statistics. The kappa statistics test analysis was addressed beyond that agreement by chance alone by computing weighted kappa and not just agreement. The receiver operating curve was prepared to determine the optimal cutoff of RUAC for the sensitivity and specificity. We used sensitivity, specificity, positive likely hood ratio, negative likely hood ratio, positive predictive value, and negative predictive value to evaluate the validity of RUAC as an alternative to MUAC which served as the reference or gold standard for acute malnutrition screening. Statistical significance was set at *p* < 0.05 and 95% confidence interval was used.

### 2.15. Operational Definition

  SAM: identified by severe wasting (WFH < −3 *z*-score for children under 5 years or MUAC < 110 mm for children 6–59 months) or the presence of bilateral pitting edema
  MAM: identified by moderate wasting (WFH < −2 *z*-score and ≥ −3 *Z*-score for children under 5 years or MUAC <120 mm and ≥110 mm for children 6–59 months)
  MUAC tape: a plastic tape which was developed by WHO to screen the nutritional status of the target groups
  MUAC: arm circumference measured at the midpoint between the tip of the shoulder and the tip of the elbow
  RUAC: arm circumference where the midpoint of the upper arm is randomly selected as opposed to MUAC
  Sensitivity: proportion of children with acute malnutrition that the test correctly identifies as malnourished
  Specificity: proportion of children without acute malnutrition that the test correctly identifies as free from acute malnutrition
  Accuracy: results of measurement close to the truth value
  Validity: measuring what it is supposed to measure of the test
  Reliability: ability to give similar result when the test is repeated
  Interpretation kappa agreement level: <0.2 = poor, 0.21–0.4 = fair, 0.41–0.6 = moderate, 0.61–0.8 = good, and 0.81–1.0 = very good [17]
  Likelihood ratio (LR): expressing how many times more (or less) likely a test result is to be found in diseased, compared with nondiseased, people
  Interpretation of +LR: >10 high probabilities, 5–10 moderate, 2–5 low probability, and 1 no change [18]
  Interpretation of −LR: <0.1 high probabilities, 0.1–0.2 moderate probabilities, 0.2–0.5 low probabilities, and 1 no change [18]
  False positive: children detected by RUAC < 12 cm but not detected by MUAC < 12 cm
  False negative: children detected by MUAC < 12 cm but not detected by RUAC < 12 cm

## 3. Results

### 3.1. Sociodemographic Characteristics of the Participants

A total of 819 children with a response rate of 100% participated in the study. The mean age (SD) of the children was 26 (14) months, ranging from 6 to 59 months. Children aged 12–23 months were highest in number. Gender distribution was slightly uneven with about 52.9% being males and 47.1% being females. The majority, 70.5% (*n* = 577), of the mothers had no formal education as compared to 47.1% (*n* = 386) of fathers. A significant number of households fall within the income range of 501–1000 (37.7%) (Table 1).

### 3.2. Feeding Practices and Nutritional Status of the Study Participants

The mean (SD) of MUAC of children was 13.34 (1.2) and the mean (SD) of RUAC was 13.40 (1.16) cm. Similarly, the MUAC measurements varied from 9 cm to 18.2 cm, and the RUAC measurements varied between 8.3 cm and 17 cm. Six hundred and ninety-six (84.8%) of the children started complementary feeding at six months of age. About 71.3%, 87%, and 77.9% of the children participated in growth monitoring and promotion (GMP) and community health day (CHD) and received counseling services during CHDs, respectively. The corresponding prevalence of acute malnutrition as determined by MUAC and RUAC was 12.5% (*n* = 102) and 6.2% (*n* = 51), respectively. One hundred and two malnourished children identified by standard MUAC were linked to targeted supplementary feeding program (TSFP) and therapeutic feeding program (TFP) for treatment (Table 2).

### 3.3. Diagnostic Agreement between MUAC and RUAC

Among the 819 study participants measured for their nutritional status, only 5.5% (*n* = 45) of them were identified to have acute malnutrition by both the MUAC and the RUAC (true positives), and 86.8% (*n* = 711) were identified as normal by both the MUAC and the RUAC (true negatives). However, 0.7% (*n* = 6) and 7.0% (*n* = 57) of children were classified as false positives and false negatives, respectively.

The MUAC and the RUAC measurement techniques agreed at kappa statistics of 0.55 (*p* < 0.001). The kappa agreement between the MUAC and the RUAC measurement to detect SAM and MAM was 0.38 and 0.47, respectively. The agreement between the two measuring techniques was almost the same in the detection of malnutrition in females (*k* = 0.56) compared to males (*k* = 0.54). However, the agreement between the MUAC and the RUAC was better in detecting acute malnutrition in the children aged 24–35 months (*k* = 0.64) (Table 3). Similarly, if WHO cutoff was used, MUAC and RUAC measurement agreed at kappa statics of 0.807 with at *p* value < 0.0001 (Table 3).

### 3.4. Validity of RUAC at NCHS Cutoff

The validity of RUAC to detect acute malnutrition was tested. Although there is no gold standard to accurately detect malnutrition, an ideal anthropometric indicator, we use MUAC less than 12 cm as the golden standard measurement tool reasonably. RUAC seems to have a high probability for identifying rightly the nonmalnourished children as demonstrated by the high specificity of 99% at 95% CI (98.1–99.3). The sensitivity for RUAC however was very low 44% at 95% CI (35%–54%). The negative likelihood ratio for RUAC was close to 1 (0.56). The sensitivity of RUAC decreased from Global wasting (44%) to SAM (27.8%) and MAM (32%). The sensitivity of RUAC was the lowest (12.5%) for the 36- to 47-month-old children with 0.12 Youden index value. However, RUAC's sensitivity to identify acute malnutrition was relatively better for the age group of 12 to 23 months old (48.8%) and 24 to 35 months old (50%) (Table 4).

### 3.5. Optimal Cutoff of RUAC Measurement against Standard MUAC

**Figure 2:**
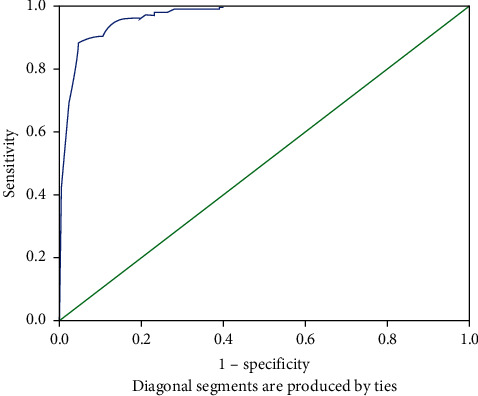
ROC curve of RUAC against standard MUAC.

A receiver operating curve (ROC) was plotted to determine the optimal cutoff and sensitivity and specificity for the RUAC and was found to be a sensitivity of 81% and specificity of 96.9%. At these optimal validity values, the cutoff for MUAC is 12.5 cm (Table 5 and Figure 2).

### 3.6. Validity of RUAC WHO 2006 Cutoff

The validity of RUAC less than 12.5 cm to detect acute malnutrition was tested using MUAC less than 12.5 cm as the standard measurement tool.  Table 6 summarizes the validity tests on the RUAC. The RUAC seems to have a high probability of identifying rightly the malnourished children as demonstrated by the high specificity (96.9%) and sensitivity (81%). However, its performance varies through age groups (Table 6).

## 4. Discussion

### 4.1. Nutritional Status of Children Based on MUAC versus RUAC

Findings from this study suggested that MUAC identified much more children with acute malnutrition compared to RUAC throughout the age range of the children. About 12.5% and 6.2% of the children below the age of 24 months were identified as having acute malnutrition by MUAC and RUAC, respectively (Table 2). Global acute malnutrition showed a similar pattern in that most of the children identified as acutely malnourished by MUAC were below the age of 24 months. MUAC was reported to be more biased to children aged 6–23 months in the detection of malnutrition in under-five children [19–21].

### 4.2. Diagnostic Agreement of MUAC and RUAC

Finding from this study revealed that the agreement level between the MUAC and RUAC based on the NCHS cutoff (< 12 cm) was moderate throughout the age groups, sex, and malnutrition level (except 24 to 35 age groups). Out of 12.5% (*n* = 102) of children who were identified as malnourished by the MUAC, only 5.5% (*n* = 45/819) of the children were identified by both indicators as malnourished (agree). Cohen's *k* was run to determine the concordance or agreement between the MUAC and the RUAC measurements and found out that the two measurement techniques agreed at kappa statics of 0.452 at *p* < 0.001. According to the interpretation of kappa [17], the level of agreement among the two measures was moderate. Thus, it is not appropriate to screen the nutritional status of children using the RUAC.

When this study stratified the RUAC and the MUAC by sex, out of 433 male children, 10.4% (*n* = 45) were detected as malnourished by MUAC (<12 cm). However, only 5.8% (*n* = 25) of male children were detected as malnourished by both methods and thus 4.6% (*n* = 20) of malnourished children were excluded by the RUAC though they were screened as malnourished by the MUAC, the standard method. On the other hand, 14.7% (*n* = 57) of female children were screened as malnourished by the MUAC (< 12 cm) but only 6.7% (*n* = 26) of the female children were classified as malnourished by the RUAC implying that 8% (*n* = 31) of malnourished female children were wrongly classified as well nourished by the RUAC.

The two measurement techniques (RUAC and MUAC) had a slightly better agreement in screening female children than male children for undernutrition (*K* = 0.562 > 0.537; *p* < 0.001), but the agreement level was still moderate for both sexes. This study also found that the agreement levels between the MUAC and the RUAC measurements were better at 24 to 35 months of age (kappa = 0.631; *p* < 0.001).

When the new WHO 2006 cutoff was used, 24.8% (*n* = 203) of children were identified by the MUAC as malnourished, whereas 22.3% (*n* = 183) of these children were detected as malnourished by both the MUAC and the RUAC, indicating a better agreement between these two measurement techniques (*K* = 0.824; *p* < 0.001). However, there was a difference in the level of agreement for both sexes (*K* = 0.811 for females and *K* = 0.802 for males). When stratified by age group, the agreement level in the 24- to 35-month age group was high (*K* = 0.938; *p* < 0.001) as compared to the rest of the age groups.

Pearson correlation coefficient was also used to evaluate the linear relationship between the two measuring techniques and the two measuring techniques had a strong positive correlation (*r* = 0.93).

### 4.3. Ability of RUAC in Detecting Acute Malnutrition in Children

Taking into consideration that the MUAC is a reasonable gold standard for acute malnutrition, the RUAC had a low sensitivity (44%) to identify children classified as malnourished by the “gold standard” using 12 cm as a cutoff. This suggests that using the RUAC may leave out many children who are malnourished out of the programs which are intended to benefit them. The purpose of community screening for malnutrition is to diagnose the condition early, but with such a high number of false negatives generated by RUAC, this goal may not be achievable using the RUAC.

The sensitivity of the RUAC in screening the children aged 36–47 months was low (12.5%) and the negative likelihood ratio (0.87) was also close to 1. Similarly, Youden index for this age group was 0.12. Such a low sensitivity of the RUAC to identify malnutrition in children aged 36–47 months shows that the RUAC may underestimate malnutrition in this age group. Moreover, the negative predictive value (NPV) of the RUAC on MAM children was low (60%). Similarly, the positive predictive values for RUAC to identify malnutrition in children aged 36–47 and 48–59 months were 50%, 95% CI (0.094–0.91), and 67%, 95% CI (0.21–0.094), respectively (Table 5). This indicates that more false-negative results are generated by RUAC which might delay early treatment of malnutrition in these children and exclude SAM cases from the stabilization center (SC). As a result, children might further deteriorate into SAM with complications and even death within some days after screening. The risk of death increases with the increase of the severity of malnutrition. However, our results indicated that the sensitivity of RUAC deteriorated with an increase in severity (MAM 32%, 95% CI (23.1–42.7) and SAM 27.8%, 95% CI (12.5–50.8)). Though the main aim of nutrition screening using the MUAC is to reach children before they got deteriorated in their nutritional status, the RUAC has failed to identify those at risk of malnutrition and ultimately failed to predict risk of death in malnourished children from our study communities.

Receiver operating characteristics curve which is a graph of sensitivity (*y*-axis) versus 1 − specificity (*x*-axis) is used in clinical epidemiology to quantify how accurately medical diagnostic tests (or systems) can discriminate between two patient states, typically referred to as “diseased” and “nondiseased,” and determine a cutoff value for a clinical test [22]. In our study, a receiver operating curve was plotted to determine the optimal cutoff for the RUAC measurement by considering standard the MUAC measurement as a reference. A statistical software found that the area under the curve is *C* = 0.97 with SE = 0.007 and 95% CI from 0.957 to 0.983 (Figure 2). The best cutoff that maximizes (sensitivity + specificity) is 12.5 cm (Table 5).

### 4.4. Appropriateness of RUAC as an Indicator of Acute Malnutrition

Though the RUAC is simple, low cost, and more acceptable to children than the MUAC steps, these properties should be considered on the condition that the indicator cannot effectively detect malnutrition. Based on the evidence presented in this study, the competence of the RUAC to effectively and uniformly detect malnutrition in children between the ages of 6–59 months is not convincing at 12 cm cutoff.

Among the notable strengths of this study were the large sample size and first effort to report the diagnostic concordance between the RUAC and the MUAC at the community level. The analysis was based on cross-sectional assessments alone with no possibility to assess the functional outcomes of the RUAC while this was useful to examine the preexisting hypotheses to explain diagnostic agreement which was one of the limitations for this study. Moreover, the findings of this study may not be applicable to all community health extension workers and health development armies doing a rapid assessment for malnutrition at the community level as our data collectors were diploma nurses.

## 5. Conclusion

In conclusion, this study revealed that the MUAC was superior to the RUAC to identify malnourished children as a tool for nutrition screening at the NCHS cutoff (12 cm) in communities where child malnutrition is a serious public health problem. However, the RUAC seems to have improved its sensitivity, specificity, and kappa agreement level when the new WHO cutoff point (< 12.5 cm) was used.

The nutrition screening for children aged less than five years in the study communities should be done using the MUAC. Hence the use of the RUAC at cutoff of 12 cm was discouraged for nutrition screening of children aged less than five years from the study communities. If the RUAC is to be used as a screening tool in the study communities, the cutoff should be adjusted to 12.5 cm. The scientific communities should conduct further studies on the treatment outcomes and length of stay children admitted in therapeutic feeding programs using the RUAC.

## Figures and Tables

**Table 1:** Sociodemographic characteristics of the study participants from Lanfro District, Silte Zone, Southern Ethiopia, 2016 (*n* = 819).

Variables		*N* (%)
Sex	Female	386 (47.1)
	Male	433 (52.9)

Age of child	6–11	146 (17.8)
	12–23	235 (28.7)
	24–35	164 (20.0)
	36–47	154 (18.8)
	48–59	120 (14.7)

Religion	Muslim	797 (97.3)
	Orthodox	19 (2.3)
	Protestant	3 (0.4)

Monthly income	<250	82 (10.0)
	251–500	180 (22.0)
	501–1000	309 (37.7)
	1001 and above	248 (30.3)

Maternal education	No formal education	577 (70.5)
	Read and/or write	95 (11.65)
	Elementary school	111 (13.6)
	High school and above	36 (4.4)

Paternal education	No formal education	386 (47.1)
	Read and/or write	176 (21.5)
	Elementary school	202 (24.7)
	High school and above	55 (6.7)

**Table 2:** Child feeding practices and nutritional status of 6–59-month-old children from Lanfro District, Silte Zone, Southern Ethiopia, 2016 (*n* = 819).

Variables		*N* (%)
Start complementary foods at six months of age	Yes	696 (84.8)
	No	123 (15)

GMP service utilization	Yes	584 (71.3)
	No	235 (28.7)

Participation in CHDs in the last three months prior to the data collection	Yes	713 (87.1)
	No	106 (12.9)

Counseled during CHDs	Yes	638 (77.9)
	No	181 (22.1)

Vitamin A capsule supplementation within the last 6 months	Yes	773 (94.4)
	No	46 (5.6)

Prevalence of acute malnutrition at NCHS cutoff (<12 cm) based on MUAC	SAM	18 (2.2)
	MAM	84 (10.3)
	GAM	102 (12.5)

Prevalence of acute malnutrition at NCHS cutoff (<12 cm) based on RUAC	SAM	6 (0.7)
	MAM	45 (5.5)
	GAM	51 (6.2)

Prevalence of acute malnutrition based on WHO cutoff (<12.5 cm) based on MUAC	SAM	33 (4.3)
	MAM	170 (24.8)
	GAM	203 (24.7)

Prevalence of acute malnutrition based on WHO cutoff (<12.5 cm) based on RUAC	SAM	31 (3.8)
	MAM	153 (18.7)
	GAM	184 (22.5)

SAM = severe acute malnutrition, MAM = moderate acute malnutrition, GAM = global acute malnutrition, CHD = community health day, and GMP = growth monitoring and promotion.

**Table 3:** Degree of agreement (kappa) between the two measurements for detecting acute malnutrition in 6–59-month-old children from Lanfro District, Southern Ethiopia, 2016 (*n* = 819).

RUAC			Kappa	*p* value
MUAC NCHS cutoff	Sex	Female	0.56	<0.001
		Male	0.54	
		Both sexes	0.55	
	Age (months)	6–11	0.53	
		12–23	0.56	
		24–35	0.64	
		36–47	0.21	
		48–59	0.48	
		Combined	0.55	
	Nutritional status	SAM	0.38	
		MAM	0.47	
		GAM	0.55	

MUAC WHO cutoff	Sex	Female	0.81	<0.001
		Male	0.80	
		Both sexes	0.81	
	Age (months)	6–11	0.76	
		12–23	0.75	
		24–35	0.94	
		36–47	0.72	
		48–59	0.79	
		Combined age group	0.81	
	Nutritional status	SAM	0.73	
		MAM	0.74	
		GAM	0.81	

**Table 4:** Validity tests on RUAC using MUAC measurement as the golden standard in 6–59-month-old children from Lanfro District, SNNPR, Southern Ethiopia, 2016 (*n* = 819).

Indicators		Sensitivity % (95% CI)	Specificity % (95% CI)	Positive likelihood ratio (95% CI)	Negative likelihood ratio (95% CI)	Youden index	PPV % (95% CI)	NPV % (95% CI)
Nutritional status	SAM	27.8 (12.5–50.9)	99 (99.3–99.9)	222.5 (27–1808.9)	0.72 (0.54–0.96)	0.26	83 (43.6–96.9)	98.4 (97.3–99)
	MAM	32 (23.1–42.7)	97.5 (96.2–98.4)	13 (7.6–22.8)	0.69 (0.6–0.8)	0.29	60 (45.4–72.9)	92 (90.594.2)
	GAM	44 (34.8–53.8)	99 (98.1–99.3)	52.7 (23.3–120.4)	0.56 (0.47–0.6)	0.43	88 (76.6–94.5)	92.5 (90.5–94.2)

Age in months	6–11	42.8 (26.5–60.9)	99 (95.4–99.8)	50.6 (6.5–372.9)	0.57 (0.42–0.79)	0.42	92 (66.7–98.6)	87.9 (87.4–92.4)
	12–23	48.8 (34.6–63.2)	97.8 (94.7–99.2)	23 (8.5–64.8)	0.52 (0.39–0.7)	0.46	84 (65.4–93.6)	89.5 (84.6–92.9)
	24–35	50 (29–70.9)	99.3 (96.2–99.9)	73 (9.1–543.1)	0.5 (0.32–0.79)	0.49	90 (59.6–98.2)	93.5 (88.5–96.5)
	36–47	12.5 (2.2–47.1)	99.2 (96.2–99.9)	18 (1.3–265.6)	0.88 (0.67–1.1)	0.12	50 (9.4–90.5)	95 (91.4–98.3)
	48–59	40 (11.8–76.9)	99 (95–99.8)	47 (5.1–437.8)	0.6 (0.96.–1.22)	0.39	66.7 (20.7–93.8)	97 (92.7–99.2)
	Combined	44 (34.8–53.8)	99 (98–99)	52.7 (23.3–120.4)	0.56 (0.47–0.6)	0.43	88 (76.6–94.5)	92.5 (90.5–94.2)

Sex	Female	43.8 (31.8–56.6)	99.7 (98.3–99.9)	144 (19.9–104.3)	0.56 (0.45–0.7)	0.43	96 (81.1–99.3)	91 (87.7–93.6)
	Male	44 (30.8–58.8)	98.7 (97–99.4)	34 (13.4–87.4)	0.56 (0.43–0.73)	0.43	80 (60.8–91.4)	93.8 (91.1–95.8)
	Combined	44 (34.8–53.8)	99 (98.1–99.1)	52.7 (23.1–120.4)	0.56 (0.47–0.6)	0.43	88 (76.6–94.5)	92.5 (90.5–94.2)

Youden index = (sensitivity + specificity) – 1. PPV: positive predictive value; NPV: negative predictive value.

**Table 5:** Performance of RUAC at different cutoff values in identifying malnourished children against MUAC from Lanfro District, Silte Zone, SNNPR 2016 (*n* = 819).

Cutoffs	Sensitivity %	Specificity %	Youden index
<11 cm	4.9	99.8	0.047
<11.5 cm	28.4	99.7	0.28
<12 cm	44.1	99.2	0.43
<12.5 cm	81.1	96.9	0.78
<13 cm	99	72.1	0.71
<13.5 cm	100	52.6	0.52
<14 cm	100	36.2	0.36
<14.5 cm	100	19.1	0.19

**Table 6:** Validity tests on RUAC measurements at WHO cutoff (<12.5 cm) from Lanfro District, SNNPR, Southern Ethiopia, 2016 (*n* = 819).

Indicators		Sensitivity % (95% CI)	Specificity % (95% CI)	Positive likelihood ratio (95% CI)	Negative likelihood ratio (95% CI)	Youden index	PPV % (95% CI)	NPV % (95% CI)
Nutritional status	SAM	72 (55.8–84.9)	99 (98.2–99.6)	81.6 (37.9–175.7)	0.28 (0.18–0.48)	0.71	77.4 (60.2–87.6)	98 (97.8–99.4)
	MAM	76.8 (69.8–82.6)	95.8 (94–97.2)	18.6 (12.7–27.2)	0.24 (0.18–0.32)	0.73	82.4 (75.5–87.6)	94.3 (92.3–95.8)
	GAM	81 (75.4–86.1)	96.9 (95.2–98.2)	26.3 (16.8–41.2)	0.19 (0.14–0.25)	0.78	89.6 (84.4–93.3)	94 (91.9–95.5)

Age in months	6–23	79.9 (70.1–80.1)	91.6 (83.6–95.8)	9.9 (4.8–20.4)	0.17 (0.09–0.3)	0.75	88 (77.8–0.94)	88 (79–93.5)
	12–23	76.7 (66.7–84.4)	95 (91.5–98)	19 (8.6–42.2)	0.24 (0.16–0.35)	0.72	91 (81.9–96)	87.7 (81.8–91.9)
	24–35	93 (78.6–98)	99 (95.8–99.8)	125 (17.7–883.4)	0.07 (0.02–0.26)	0.92	96.5 (82.5–99.4)	98 (94.7–99.5)
	36–47	70.6 (46.8–86.7)	97.8 (93.7–99)	32 (10–102.8)	0.3 (0.14–0.63)	0.68	80 (54.8–92.9)	96.4 (91.8–98.4)
	48–59	85.7 (48.7–97.4)	98.5 (93.7–99.5)	48 (11.8–197.7)	0.15 (0.02–0.89)	0.84	75 (40.9–92.8)	98 (94.7–99.5)
	Combined	81.2 (75.4–86.1)	96.9 (95.2–98.2)	26.3 (16.8–41.2)	0.19 (0.14–0.25)	0.78	89.6 (84.4–93.3)	94 (91.9–95.5)

Sex	Female	81 (72.2–87.5)	97 (94.5–98.6)	28.9 (14.52–57.7)	0.20 (0.13–0.29)	0.78	91 (83.3–95.4)	93 (90.2–95.8)
	Male	81.5 (72.9–87.8)	97 (94.1–98.1)	24.5 (13.5–44.1)	0.19 (0.1–0.28)	0.79	88 (80.4–93.4)	94.4 (10.6–26.5)
	Combined	81.2 (75.4–86.1)	96.9 (95.2–98.2)	26.3 (16.8–41.2)	0.19 (0.14–0.25)	0.78	89.6 (84.4–93.3)	94 (91.9–95.5)

Youden index = (sensitivity + specificity) – 1. PPV: positive predictive value; NPV: negative predictive value.

## Data Availability

The data sets analyzed during the current study are available from the corresponding author upon reasonable request.

## Abbreviations

CI: Confidence interval
ENA: Emergency nutrition assessment
GAM: Global acute malnutrition
MAM: Moderate acute malnutrition
MUAC: Mid upper arm circumference
NCHS: National Center for Health Statistics
NPV: Negative predictive value
RUAC: Random upper arm circumference
SC: Stabilization center
SAM: Severe acute malnutrition
ENA for SMART: Emergency nutrition assessment for standardized monitoring and assessment of relief and transitions
SNNPR: Southern Nations, Nationalities, and Peoples' Region
TSFP: Targeted supplementary feeding program
TFP: Therapeutic feeding program
WHO: World Health Organization
UNICF: United Nations Children's Fund.

## Supplementary Materials

Supplementary Materials Table S1: diagnostic test characteristics and their definitions.
