# Supplementary material for: Diagnostic Concordance between Random Upper Arm Circumference and Mid Upper Arm Circumference Measurements among Children Aged 6–59 Months in South Ethiopia: A Community-Based Cross-Sectional Study
Source: J Nutr Metab. 2021 Mar 31;2021:6654817. doi: 10.1155/2021/6654817 (PMC8032539; doi:10.1155/2021/6654817)
Supplement: Supplementary Materials — Table S1: diagnostic test characteristics and their definitions. [file 6654817.f1.docx]

Table S1: Diagnostic test characteristics and their definitions

| Test characteristic | Definition |
| --- | --- |
| Sensitivity | The ability of a test to correctly identify those with disease. Sn = true positive/(true positive + false negative) |
| Specificity | The ability of a test to correctly identify those without disease. Sp = true negative/(true negative + false positive) |
| Positive predictive value | The probability that a person with a positive test truly has disease. PPV = true positive/(true positive + false positive) |
| Negative predictive value | The probability that a person with a negative test is truly Disease free. NPV = true negative/(true negative + false negative) |
| Likelihood ratio positive | The ratio of the probability of a positive test in those with disease divided by the probability of a positive test in those Without disease. LR+ = sensitivity/(1 – specificity) |
| Likelihood ratio negative | The probability of a negative test in those with disease divided by the probability of a negative test in those without disease. LR- = (1-sensitivity)/specificity |
| Simplicity | The method can be easily administered by non-clinicians |
| Acceptability | The method is acceptable to the subject and others |
| Precision | The degree of reproducibility among independent measurements of the same true value (also known as reliability). |
